# The impact of single nucleotide polymorphisms on return-to-work after taxane-based chemotherapy in breast cancer

**DOI:** 10.1007/s00280-022-04499-z

**Published:** 2023-01-04

**Authors:** Cathrine F. Hjorth, Per Damkier, Tore B. Stage, Søren Feddersen, Stephen Hamilton-Dutoit, Bent Ejlertsen, Timothy L. Lash, Henrik Bøggild, Henrik T. Sørensen, Deirdre Cronin-Fenton

**Affiliations:** 1 Department of Epidemiology, Department of Clinical Medicine, Aarhus University Hospital, Aarhus University, Aarhus, Denmark; 2 Department of Clinical Pharmacology, Odense University Hospital, Odense, Denmark; 3 Department of Clinical Research, University of Southern Denmark, Odense, Denmark; 4 Department of Public Health, University of Southern Denmark, Odense, Denmark; 5 Clinical Pharmacology, Pharmacy and Environmental Medicine, Department of Public Health, University of Southern Denmark, Odense, Denmark; 6 Department of Clinical Biochemistry, Odense University Hospital, Odense, Denmark; 7 Department of Pathology, Department of Clinical Medicine, Aarhus University Hospital, Aarhus University, Aarhus, Denmark; 8 Department of Oncology, Rigshospitalet, Copenhagen University, Copenhagen, Denmark; 9 Danish Breast Cancer Group, Rigshospitalet, Copenhagen University Hospital, Copenhagen, Denmark; 10 Department of Epidemiology, Rollins School of Public Health, Emory University, Atlanta, GA USA; 11 Public Health and Epidemiology Group, Department of Health Science and Technology, Aalborg University, Aalborg, Denmark; 12 Unit of Clinical Biostatistics, Aalborg University Hospital, Aalborg, Denmark

**Keywords:** Single nucleotide polymorphisms, Taxane, Docetaxel, Breast neoplasms, Cohort study, Return-to-work

## Abstract

**Purpose:**

Breast cancer treatment is associated with adverse effects, which may delay return-to-work. Single nucleotide polymorphisms (SNPs) may influence the risk and severity of treatment toxicities, which in turn could delay return-to-work. We examined the association of 26 SNPs with return-to-work in premenopausal women with breast cancer.

**Methods:**

Using Danish registries, we identified premenopausal women diagnosed with non-distant metastatic breast cancer during 2007‒2011, assigned adjuvant combination chemotherapy including cyclophosphamide and docetaxel. We genotyped 26 SNPs in 20 genes (*ABCB1, ABCC2, ABCG2, CYP1A1, CYP1B1, CYP3A, CYP3A4, CYP3A5, GSTP1, SLCO1B1, SLCO1B3, ARHGEF10, EPHA4, EPHA5, EPHA6, EPHA8, ERCC1, ERCC2, FGD4 and TRPV1)* using TaqMan assays. We computed the cumulative incidence of return-to-work (defined as 4 consecutive weeks of work) up to 10 years after surgery, treating death and retirement as competing events and fitted cause-specific Cox regression models to estimate crude hazard ratios (HRs) and 95% confidence intervals (CIs) of return-to-work. We also examined stable labor market attachment (defined as 12 consecutive weeks of work).

**Results:**

We included 1,964 women. No associations were found for 25 SNPs. The cumulative incidence of return-to-work varied by *CYP3A5* rs776746 genotype. From 6 months to 10 years after surgery, return-to-work increased from 25 to 94% in wildtypes (*n* = 1600), from 17 to 94% in heterozygotes (*n* = 249), and from 7 to 82% in homozygotes (*n* = 15). The HR showed delayed return-to-work in *CYP3A5* rs776746 homozygotes throughout follow-up (0.48, 95% CI 0.26, 0.86), compared with wildtypes. Estimates were similar for stable labor market attachment.

**Conclusion:**

Overall, the SNPs examined in the study did not influence return-to-work or stable labor market attachment after breast cancer in premenopausal women. Our findings did suggest that the outcomes were delayed in homozygote carriers of *CYP3A5* rs776746, though the number of homozygotes was low.

**Supplementary Information:**

The online version contains supplementary material available at 10.1007/s00280-022-04499-z.

## Introduction

Advances in breast cancer diagnosis and treatment have enlarged the pool of breast cancer survivors [1], emphasizing the need to better understand breast cancer survivorship. Return-to-work may be a marker of recovery and return to daily activities after concluding breast cancer treatment [2].

Up to 80% of women with breast cancer return-to-work during or after adjuvant treatment, but some may have delayed or may never return-to-work [3–5]. Return-to-work after breast cancer may be hindered by the type of work involved and by the working environment, but may also be affected by survivor well-being, health and functional impairment, societal factors, socioeconomic position, and family support [6, 7]. Research suggests that chemotherapy may impede return-to-work in breast cancer survivors, probably due to adverse effects during or after chemotherapy [5], this may be affected by socioeconomic position.

Premenopausal women receive taxane-based adjuvant chemotherapy as guideline treatment [8]. In the absence of their cancer, these women are likely to contribute substantial person-years to the workforce [9]. While improving survival [10], taxanes induce a number of potentially severe adverse effects [11–13]. Single nucleotide polymorphisms (SNPs) in genes related to taxane transport, drug metabolism, neural function/repair or DNA-repair mechanisms have been linked to increased risks of severe adverse effects, including chemotherapy-induced peripheral neuropathy [14–17]. Accordingly, such SNPs may be associated with slower recovery and delayed return to normal daily activities, including work. Tamoxifen treatment, given to premenopausal women with estrogen receptor (ER) positive tumors, may modify these associations [18]. Tamoxifen shares metabolizers and transporters with taxanes, and is associated with adverse effects [18, 19], which may also influence return-to-work.

No studies have explored whether SNPs connected to taxane effectiveness or adverse effects influence return-to-work after cancer. Therefore, we examined this in premenopausal breast cancer patients treated with taxane-based chemotherapy. Furthermore, we examined the mediating role of estrogen receptor (ER) status and indicators of socioeconomic position.

## Materials and methods

### Data sources

Denmark has a tax-supported population-wide health care system [20]. We linked individual-level electronic data from Danish administrative and medical registries with biological data using a unique ten-digit personal identifier assigned to all Danish residents at birth or immigration [21]. The Danish Breast Cancer Group (DBCG) registers all incident breast cancers, along with clinical information and follow-up data on recurrences and other malignancies [22]. The Danish National Pathology Registry [23] has routinely recorded information on all histopathological analyses and the whereabouts of associated formalin-fixed paraffin-embedded (FFPE) tissue blocks. The Cause of Death Registry records date of death along with underlying and contributory causes registered by the inspecting doctor [24]. In addition, we summarized comorbidities via the Charlson Comorbidity Index using diagnoses identified in the Danish National Patient Registry [25]. Information on childbirths after breast cancer diagnosis was collected from the Danish Medical Birth Registry [26]. Highest attained education level at date of breast cancer diagnosis was collected from the Danish Population’s Education Registry [27], household income from the Danish Income Statistics Registry [28], and cohabitation status from Danish Civil Registration System [20].

The Danish labor market model, also known as the *flexicurity* model, favors employers with flexible hiring and firing rules, while it safeguards employees with a generous social system and security net [29]. The Danish state provides substantial subsistence payments, unemployment benefits, and a range of social and health-related benefits. Since 1991, these payments have been registered on a weekly basis in the Danish Register for Evaluation of Marginalization (DREAM) [30]. During the study period (see below), the length of employer-paid sick leave ranged between 14 and 31 days; employer-paid sick leave is not registered in DREAM. Assuming breast cancer patients undergoing surgery and chemotherapy have longer periods of sick leave, DREAM can capture the length of absence from the labor market, and hence return-to-work.

### Study cohort

Our study cohort was nested in the ProBe CaRe (Predictors of Breast Cancer Recurrence) cohort [31]. This cohort includes premenopausal women diagnosed with incident non-distant metastatic breast cancer in Denmark during 2002–2011 (*n* = 5959), registered in DBCG. We restricted to women who were diagnosed during 2007–2011, during which period most premenopausal breast cancer patients were recommended three cycles of epirubicin and cyclophosphamide every third week, followed by three cycles of docetaxel, while some received sequential docetaxel and cyclophosphamide [32]. We included the women who at diagnosis were aged ≤ 55 years, had chemotherapy as intended adjuvant treatment, and were employed any time during the 2 months before breast cancer primary surgery. We excluded women who were on maternity leave during the week of surgery (Supplemental Fig. S1). We expected all women to be not working at least 1 day during the week of surgery regardless of any payouts.

### Tumor specimens and genotyping

Procedures for FFPE collection, tumor tissue procurement, and DNA extraction have been described previously [18]. We selected 26 candidate SNPs in 20 genes related to taxane transport (*ABCB1, ABCC2, ABCG2, SLCO1B1, SLCO1B3*), metabolism (*CYP1A1, CYP1B1, CYP3A, CYP3A4, CYP3A5, GSTP1*), DNA repair (*ERCC1, ERCC2*), and SNPs associated with neural function or repair (*EPHA4, EPHA5, EPHA6, EPHA8, FGD4, ARHGEF10, TRVF1*).

Seven SNPs were genotyped in a previous project [18] and nineteen SNPs were genotyped for this project using commercially available TaqMan assays on a StepOne Plus real-time instrument (Applied Biosystems, Thermo Fisher Scientific, Foster City, California, USA). Genotyping was performed using 2 µL genomic DNA (10 ng/µL) extracted from FFPE tissue, 5 µL TaqMan Genotyping Master Mix, and 0.5 µL TaqMan allelic discrimination assay (VIC- and FAM-labeled probes) in a final volume of 10 µL. Thermal cycling conditions were: 95 °C for 10 min followed by 50 cycles of 95 °C for 15-s, and 60 °C for 60-s. Genotype calling was performed using the QuantStudio Software V1.3 with automatic calling. After automatic calling genotype results were manually inspected, acceptance was overridden manually if irregular amplification curves were observed. We compared the observed genotype frequencies with those expected under Hardy–Weinberg equilibrium (HWE), and allele frequencies with those reported in European non-Finnish female populations reported in the Genome Aggregation Database (gnomAD) [33].

### Outcomes

We assumed the women were working if they did not receive any social benefits, as done in other studies [30, 34–37]. We also included women receiving substituted unemployment benefits for part-time work or educational grants (see coding list, Supplemental Table S1). We defined return-to-work as 4 consecutive weeks of work. We examined stable labor market attachment defined as 12 consecutive weeks of work.

### Covariates

Patient, tumor, and treatment characteristics included age group, comorbidities, education level, cohabitation/marital status, household income, ER status combined with endocrine therapy, double/triple negative tumors, TNM stage [38], grade (in ductal and lobular tumors), surgery type, and intended radiotherapy. Detailed categorizations of the covariates are listed in Supplemental Tables S1 and S2.

### Statistical analyses

We examined the cumulative incidence of return-to-work and stable labor market attachment using the Nelson–Aalen estimator, treating death and retirements as competing risks [39]. To examine time to return-to-work and stable labor market attachment, we fitted cause-specific Cox regression models to calculate crude hazard ratios (HRs) and associated 95% confidence intervals (CIs) of return-to-work and stable labor market attachment by genotypes within time periods all counting from day of surgery. Follow-up continued until return-to-work or stable labor market attachment or until maternity leave, childbirth, recurrence, death, emigration, other malignancies, early or normal retirement, or 25th September 2017. All statistical analyses were conducted using SAS software (Cary, NC).

### Additional analyses

We examined effect-measure modification stratifying the univariate models by ER/endocrine therapy status, income, education level and cohabitation/marital status. We performed several sensitivity analyses by alternative pre-surgery employment criteria: narrowing the assessment window to 4 weeks pre-surgery and applying a stricter criterium of at least 4 weeks of employment up to 2 months before surgery. Breast cancer survivors with physical or psychological sequelae may qualify for a flexible job schedule [40]. We, therefore, reran analyses including flexible job schedules in the return-to-work outcome. As suggested by others [41], we stratified our assessment of CYP3A4 by CYP3A5 genotype, considering any variant carriers versus wildtype.

## Results

The ProBe CaRe cohort included a total of 5,959 premenopausal women. After exclusions, our final analytic cohort consisted of 1,964 women (Supplemental Fig. 1). The majority were aged 45–55 years (57%, median age: 46, interquartile range 41–49), had no diagnosed comorbidities (89%), were cohabiting (79%), were educated at intermediate level (48%), and belonged to the high-income group (63%). Most tumors were ER + (79%), stage II (57%), and 11% were TNBC (Table 1).

**Table 1:** Patient, tumor, and treatment characteristics of premenopausal women diagnosed with non-distant metastatic breast cancer in Denmark during 2007–2011 assigned taxane-based chemotherapy

	Median	IQR
Age at diagnosis	46	41–49

	*N*	%
Age group at diagnosis		
< 35	129	7
35–44	724	37
45–55	1111	57
ER status		
ER–	422	21
ER +	1542	79
HER2 status		
Negative	1461	74
Positive	365	19
Not tested	138	7
Triple negative breast cancer		
No	1671	85
Yes	214	11
Not tested	79	4
TNM stage		
Stage I	502	26
Stage II	1127	57
Stage III	323	16
Missing	12	1
Histological grade		
Grade 1	292	15
Grade 2	831	42
Grade 3	643	33
Not graded	176	9
Missing	22	1
Comorbidity		
None	1741	89
1–2	166	8
3 or more	57	3
Surgery type		
Mastectomy	744	38
Lumbectomy incl. ITT Radiotherapy	< = 1225	
Missing	< 5	
Cohabitation/marital status		
Cohabiting/married	1542	79
Living alone	409	21
Missing	13	1
Income		
< Median	712	36
≥ Median	1247	63
Missing	5	0
Educational level		
Short	258	13
Intermediate	951	48
Long	735	37
Missing	20	1

*ER* estrogen receptor, *HER2* human epidermal growth factor receptor 2, *IQR* interquartile range, *ITT* intention-to-treat, *TNM* tumor node metastasis

We included 21 SNPs in the analyses, 5 SNPs were excluded due to call rates of below 95% (*ABCB1* rs10248420, *CYP1A1* rs1048943, *TRPV1* rs879207, *ARHGEF10* rs9657362, and *EPHA8* rs209709). Detailed genotyping information can be found in Supplemental Table S3.

**Fig. 1:**
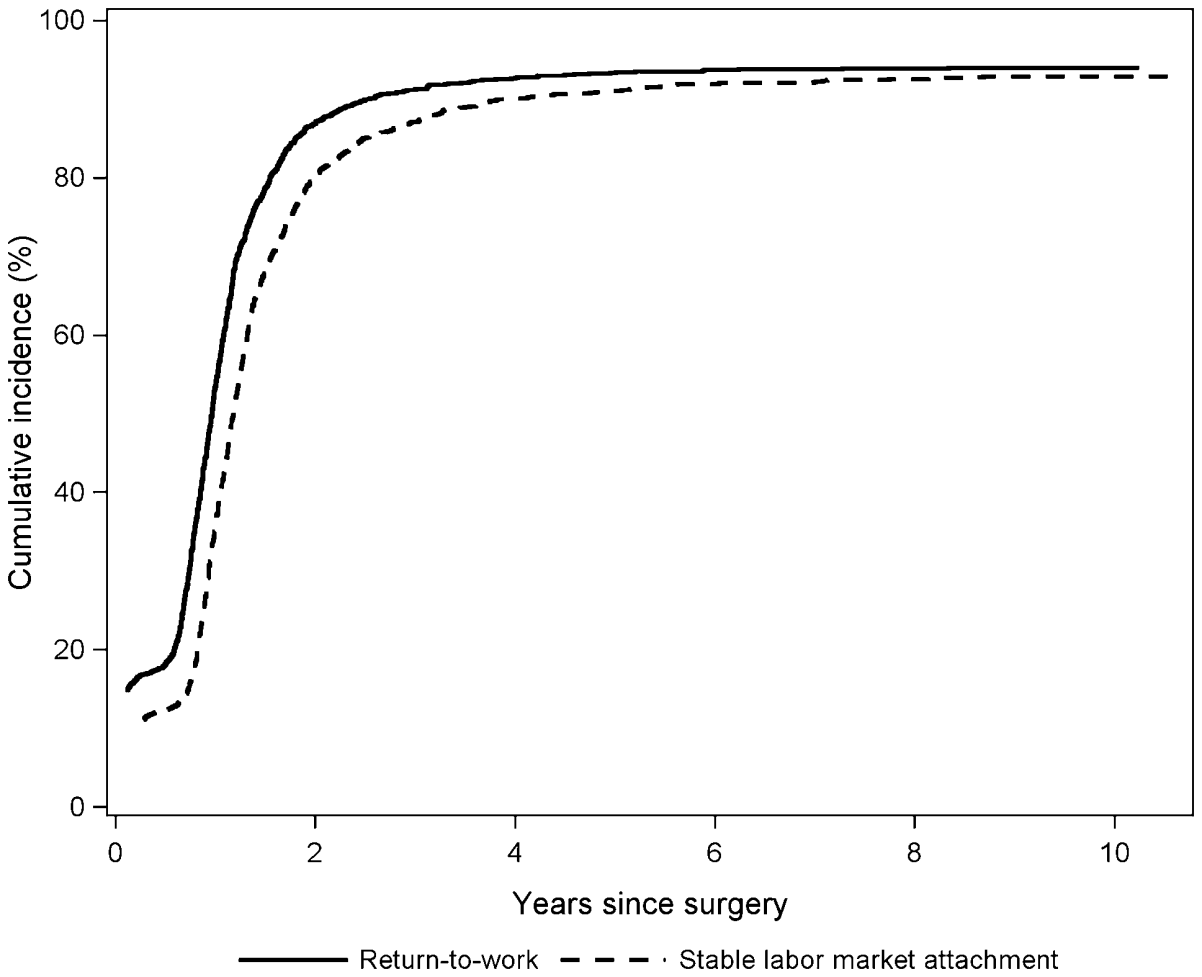
Cumulative incidence of return-to-work and stable labor market attachment

Figure 1 shows the cumulative incidences of return-to-work and stable labor market attachment, respectively, of 18% and 12% 6 months after breast cancer diagnosis, 53% and 35% 1 year after, 87% and 80% 2 years after, and 94% and 93% 10 years after.

**Fig. 2:**
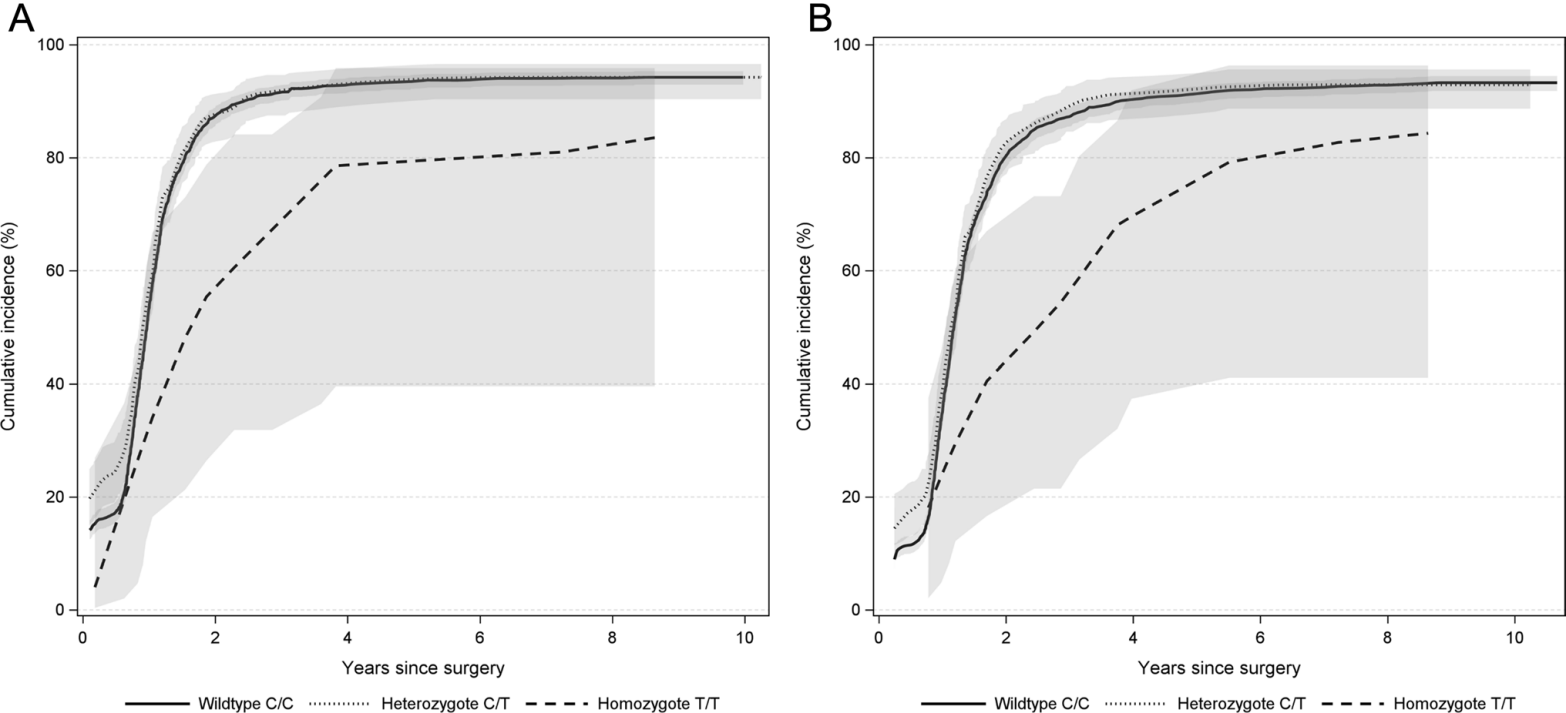
Cumulative incidence of return-to-work (**A**) and stable labor market attachment (**B**) by *CYP3A5* genotype. Curves were smoothed using loess function. The shaded bands represent associated 95% confidence intervals

The cumulative incidence of return-to-work was lower in *CYP3A5* rs776746 homozygotes (*n* = 15) than seen in wildtypes (*n* = 1600) and heterozygotes (*n* = 249). Illustrated in Fig. 2A, the cumulative incidence of return-to-work in wildtypes increased from 25% at 6 months to 94% at 10 years, in heterozygotes from 17 to 94%, and in homozygotes from 7% at 6 months and 82% at 10 years. We observed a similar delay in the cumulative incidence of stable labor market attachment among homozygotes (Fig. 2B).

**Fig. 3:**
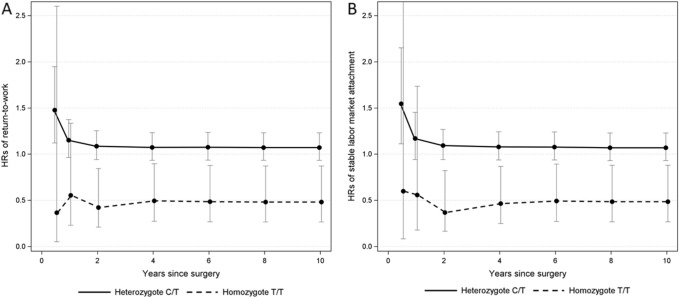
Hazard ratios with 95% CI of return-to-work (**A**) and stable labor market attachment (**B**) in *CYP3A5* rs776746 heterozygotes and homozygotes, compared with wildtypes

HRs showed delayed return-to-work and stable labor market attachment in *CYP3A5* rs776746 homozygotes compared with wildtypes throughout follow-up (Fig. 3) of approximately 50% (10-year HRs: 0.48, 95% CI 0.27–0.87 and 0.49, 95% CI 0.27–0.88, respectively).

We observed associations for other SNPs (see Supplemental Table S4), but these had limited numbers of homozygotes, and inconsistencies between cumulative incidence curves and HRs suggesting these were probably chance findings.

Although based on small strata, we observed no effect-measure modification by ER status or socioeconomic position. In the analyses factoring in flexible job schedules, the cumulative incidence of return-to-work reached 97%, but relative estimates were similar in all sensitivity analyses.

## Discussion

In this study, premenopausal women had a high cumulative incidence of return-to-work and stable labor market attachment, reaching 94% and 93% at 10 years after treatment for early breast cancer. These levels were reached already within 3–4 years. Homozygote carriers of *CYP3A5* rs776746 had delayed return-to-work and stable labor market attachment, compared with wildtypes.

Our overall assessment of return-to-work extends previous research. Arndt et al. [4] studied the cumulative incidence of return-to-work among 1,070 women with breast cancer living in Germany. Compared with our findings, they reported a slightly lower 10-year cumulative incidence of 85%, presumably due to their older study cohort including postmenopausal breast cancer survivors. Their study was prone to selection bias as they only included 5-year survivors, and therefore could have overestimated return-to-work as women dying within 5 years after surgery were then excluded. However, the higher incidence of return-to-work in our study may reflect long work life expectancy in premenopausal women and successful rehabilitation.

We investigated genes that theoretically could influence treatment effectiveness/adverse effects or that previously had been associated with such. The lack of association of the SNPs with return-to-work is encouraging for patients and contrasts with some other studies. Kus et al. [17] investigated 219 Turkish breast cancer patients and found that the *CYP3A4* rs2740574 variant allele was associated with lower risk of neuropathy, especially for chemotherapy-induced peripheral neuropathies that interfered with activities of daily living. We note that our HRs for *CYP3A4* rs2740574 were somewhat consistent with increased return-to-work, but increased return-to-work was not evident in our cumulative incidence curves. Furthermore, the Turkish study had a substantially higher frequency of *CYP3A4* rs2740574 homozygotes of 42% compared with less than 1% in our Danish cohort. A study by Eckhoff et al. [16] of 150 Danish early stage breast cancer patients, and thus similar allele frequencies to our population, found that *GSTP1* rs1138272 variant carriers had increased risk of chemotherapy-induced peripheral neuropathy during docetaxel treatment, also when examining neuropathies graded ≥ 2 [5]. Still, we found no evidence of an influence of *GSTP1* on return-to-work.

Our findings of delayed return-to-work in *CYP3A5* rs776746 homozygote women may indicate poorer recovery compared with their wildtype counterparts. CYP3A5 is a phase 1 enzyme involved in the metabolism of docetaxel in the liver. *CYP3A5* rs776746 is highly polymorphic and can cause splicing defects of mRNA. Most Caucasians are CYP3A5 non-expressors (wildtypes), while heterozygotes and homozygotes are CYP3A5 expressors [41]. As such, expressors could be expected to have higher drug clearance. This has been found in one study including Caucasian cancer patients (27% of whom had breast cancer) treated with docetaxel [41]. The *CYP3A5* non-expressing variant has been associated with reduced risk of neurotoxicity during treatment in 118 Spanish cancer patients (1/3 breast cancer) treated with paclitaxel [42], corresponding to an increased risk in expressors. Although the Spanish study did not examine long-term adverse effects, these findings could potentially explain our observed delayed return-to-work in *CYP3A5* expressors in our study. Studies on chemotherapy-induced peripheral neuropathy in taxanes suggest that the symptoms during therapy resolve or diminish after the end of treatment [43, 44]. Still, some symptoms may persist [43]. Our findings may indicate longer term adverse effects in *CYP3A5* rs776746 homozygotes. Nonetheless, only 15 women in our cohort were homozygotes for *CYP3A5* rs776746; therefore, our findings may also be attributable to chance.

The major strength of our population-based study was the nationwide genotyping of premenopausal breast cancer survivors, and the linkage to validated clinical and individual data with high completeness [20, 27, 30, 45–47]. We incorporated several quality-control measures to ensure high-quality SNP data. We did not only rely on Hardy–Weinberg disequilibrium, as this can be influenced by sample size [48, 49]. Instead, we inspected (and present) congruence between observed and expected frequencies. We excluded SNPs with call rates < 95% and with overlapping genotype clusters. DNA was derived from FFPE tumor-infiltrated tissue, which previously has been proven suitable for studies of breast cancer prognosis [50]. Moreover, studies report high genotype concordance between FFPE breast tumors and both FFPE normal lymph nodes [51, 52] and plasma [51].

Some limitations must be considered. We had no information on adverse effects and individual information on administered chemotherapy, including docetaxel plasma concentrations or cumulative dose. Adverse effects are poorly registered in Danish health registries, and chemotherapy dose capping could have been associated with treatment toxicity. Information on treatment toxicity and later adverse effects could have substantiated our interpretation but are unlikely to confound our estimates. None of the women in our study were intended to receive docetaxel monotherapy. Our findings could, therefore, be influenced by interaction as *CYP3A5* is also involved in cyclophosphamide metabolism [53].

Despite high validity of social benefit payments registered in DREAM [30, 47], this database has its limitations. Our outcomes relied on the assumption that no evidence of a payment record was equivalent to employment. Validated against self-reporting, self-supportiveness (defined by no DREAM entry, student grant, leave-of-absence schemes including maternity leave) has a positive predictive value of 98% [30]. However, this may not always indicate employment as withdrawal from the work force could be supported by savings or spouse earnings. A Danish study examining income changes after breast cancer found that those married had a lower income up to 9 years after diagnosis, while this was 6 years for those who were single [54]. This suggests that married breast cancer survivors are supported economically by their spouse.

In a study examining return-to-work after maternity leave, we validated employment (defined as no payment, or vacation from employment payouts) against records of salary payments and found an agreement of 94% (unpublished). It is likely that some women choose not to return-to-work and also avoid the bureaucracy associated with registering for social benefit payments from the public sector. In that case, we may have overestimated the cumulative incidence of return-to-work.

Our study provides novel insights that argue for more research on the impact of *CYP3A5* rs776746 on recovery in women treated with taxanes and cyclophosphamide. Such research could help identify women at risk of poor recovery after taxane-based chemotherapy. As we only examined the associations of single SNPs, future studies should include Bayesian pathway analysis considering the entire complex metabolic pathway of docetaxel [18, 55].

## Conclusion

In this population-based cohort of premenopausal breast cancer survivors with non-distant metastatic breast cancer, homozygous carriers of *CYP3A5* rs776746 had delayed return-to-work and stable labor market attachment after breast cancer. These associations—and their underlying mechanisms—need to be investigated further. Still, if validated elsewhere, these findings may indicate the utility of *CYP3A5* rs776746 to identify women at risk of a poor clinical course, who may benefit from enhanced supportive care during treatment and follow-up.

## Supplementary Information

Below is the link to the electronic supplementary material.

Supplementary file1 (DOCX 67 KB)

## Data Availability

The dataset generated and analyzed during the current study are not publicly available due to Danish legislations but are available from the corresponding author and Statistics Denmark on reasonable request and permissions from the below mentioned third parties. All the data used for the current study are stored at Statistics Denmark. Researchers can apply to Statistics Denmark for data access, conditional on obtained permissions from the DBCG, the Danish Health Authorities, the Danish Data Protection Agency, and the Central Jutland Region Committee on Health Research Ethics.
