# Supplementary material for: The impact of single nucleotide polymorphisms on return-to-work after taxane-based chemotherapy in breast cancer
Source: Cancer Chemother Pharmacol. 2023 Jan 4;91(2):157–65. doi: 10.1007/s00280-022-04499-z (PMC9905159; doi:10.1007/s00280-022-04499-z)
Supplement: Supplementary file 1 — Supplementary file1 (DOCX 67 KB) [file 280_2022_4499_MOESM1_ESM.docx]

**Supplemental material**

The impact of single nucleotide polymorphisms on return-to-work after taxane-based chemotherapy in breast cancer

**Contents**

[Figure S1. Flow diagram 2](#_Toc100051802)

[Table S1. Codelist for covariates 3](#_Toc100051803)

[Table S2. Algorithm for Charlson Comorbidity Index 4](#_Toc100051804)

[Table S3. SNP genotyping information, including observed and expected counts. 5](#_Toc100051805)

[Table S4. Hazard ratios of return-to-work and stable labor market attachment 6](#_Toc100051807)

# Figure S1. Flow diagram, study cohort.

| Source population  All women in the ProBe CaRe cohort  n=5,959 | | | | |  |
| --- | --- | --- | --- | --- | --- |
|  |  |  | |  |  |
|  |  |  | | Women diagnosed before 2007  n=2,980 | |
|  |  |  | |  |  |
|  |  |  | |  |  |
|  | |  | | Women aged >55 years  n=30 | |
|  |  |  |  |  |  |
|  |  |  |  |  |  |
|  |  |  |  | Women not assigned ITT chemotherapy  n=131 | |
|  |  |  |  |  |  |
|  |  |  | |  |  |
|  |  |  | | Women with no available tumor tissue  n=370 | |
|  |  |  | |  |  |
|  |  |  | |  |  |
|  |  |  | | Women not employed (n=424) 0–2 months* before breast cancer diagnosis and women on maternity leave at diagnosis or censoring at date of diagnosis (n=60)  n= 465 | |
|  |  |  | |  |  |
|  |  |  | |  |  |
| Study cohort  N= 1,964 | | | | |  |

* At least one week of employment during the two months preceding breast cancer surgery was required. This allowed short-term sick-leaves pre-surgery. Compared with the employed women, the women excluded because of unemployment were more often diagnosed with stage ІІІ (22% vs. 16%) and to receive a mastectomy (44% vs. 38%), to be living alone (35% vs. 21%), to be lower educated (35% vs. 13%) and have income below the source population median (66% vs. 36%). Median age and interquartile ranges (IQR) of the two group were 45 years (IQR: 41-48) and 46 years (41-49), respectively.

# Table S1. Codelist for covariates

| **Covariate** | **Definition** | **Data source and variable** |
| --- | --- | --- |
| Age group | Age at date of diagnosis | DBCG variable age |
| ER status | Until 1. July 2010 tumors were coded as ER– if ER expression was less than 10%, and ER+ if 10% or larger. Onwards, the limit was 1%. | DBCG. We used the variables p72, p73, p68 and p69. |
| HER2 status |  | DBCG: P137 |
| Triple negative breast cancers | Categorized as triple negative breast cancers if tumors were ER–, HER2–, and had negative or missing progesterone receptor status. Until 1. July 2010 tumors were coded as ER–/PR– if ER/PR expression was less than 10%, and ER+/PR+ if 10% or larger. Onwards, the limit was 1%. | DBCG: abovementioned codes and PR status using p70, p71, p74, p75. |
| Pathological stage | According to the TNM staging system | DBCG: p19 and p25 |
| Anaplastic grade | Grade 1–3 assigned ductal and lobular tumors. Others were not graded. | DBCG: P50, p51, p52 and p63a |
| Surgery type | Mastectomy or lumpectomy incl. ITT radiotherapy. | DBCG: M45 and iRT. |
| Recurrence | Date of diagnosed recurrence | DBCG: Op5simpel, op2, op3, op4 |
| Other malignancy | Date of other diagnoses of malignancies. | DBCG: Op5simpel, op2, op3, op4 |
| Childbirth | Date of childbirth | Danish Medical Birth Registry: Variable FOEDSELSDATO |
| Comorbidities | Categorized according score in the Charlson comorbidity Index. | The algorithm for CCI was based on codes in the International Classification of Diseases, Tenth Revision derived from The Danish National Patient Registry. Se algorithm is inserted in below Table S2. |
| Cohabitation | Cohabiting/maried or living alone | Based on an algorithm by Statistics Denmark (variable: familie_type) categorizing people as cohabiting if being 1) married, 2) registered partnership, 3) same address as person with common child, 4) if living with one person of the opposite sex and age difference is >15 years, no children, and living at the same address. The variable is updated annually in November. We collected the status the year preceding the year of breast cancer. |
| Education | Short education: ISCED 0–2 corresponding to <10 years of education. Intermediate education: ISCED 3–5 corresponding to >10 to 15 years of education. Long education: ISCED 6–8 corresponding to >15 years of education. | Danish Population’s Education Registry^1^ held by Statistics Denmark. We used the variable “hfaudd”, which specifies the highest attained education within a given period. We used the hf_vfra variable to identify highest attained education at date of breast cancer diagnosis. |
| Household income | Categorized using the median household income in the source population as cut-off between <median and ≥median. | Based on the variable AEKVIVADISP_13 recorded in the Danish Income Statistics Registry^2^ by Statistics Denmark. The variable includes household income after taxes, corrected for number of persons in the family. We collected household income in the two years preceding the year of breast cancer diagnosis and calculated the average. |
| Employment status |  | The DREAM database. Using the variables y_0601-y_1739 we did following categorizations:  Employment: [No Entry], 512, 651, 652, 661, 662, 794, 112, 113, 115, 112, 123, 413.  Maternity leave: 881.  Early and normal retirement: 611, 621, 622, 781, 783, 784, 793, 998. |

Abbreviations: CCI= Charlson comorbidity Index, DBCG= Danish Breast Cancer Group, ER= Estrogen receptor, HER2= Human epidermal growth factor receptor 2, ISCED= International Standard Classification of Education, ITT= Intention-to-treat, PR= progesterone, TNM= Tumor node metastasis

# Table S2. Algorithm for Charlson Comorbidity Index according to codes in the International Classification of Diseases, Tenth Revision (ICD-10).

|  | **Diseases** | **ICD-10** | **Score** |
| --- | --- | --- | --- |
| 1 | Myocardial infarction | I21;I22;I23 | 1 |
| 2 | Congestive heart failure | I50; I11.0; I13.0; I13.2 | 1 |
| 3 | Peripheral vascular disease | I70; I71; I72; I73; I74; I77 | 1 |
| 4 | Cerebrovascular disease | I60-I69; G45; G46 | 1 |
| 5 | Dementia | F00-F03; F05.1; G30 | 1 |
| 6 | Chronic pulmonary disease | J40-J47; J60-J67; J68.4; J70.1; J70.3; J84.1; J92.0; J96.1; J98.2; J98.3 | 1 |
| 7 | Connective tissue disease | M05; M06; M08; M09; M30; M31; M32; M33; M34; M35; M36; D86 | 1 |
| 8 | Ulcer disease | K22.1; K25-K28 | 1 |
| 9 | Mild liver disease | B18; K70.0-K70.3; K70.9; K71; K73; K74; K76.0 | 1 |
| 10 | Diabetes type1  Diabetes type2 | E10.0, E10.1; E10.9  E11.0; E11.1; E11.9 | 1 |
| 11 | Hemiplegia | G81; G82 | 2 |
| 12 | Moderate to severe renal disease | I12; I13; N00-N05; N07; N11; N14; N17-N19; Q61 | 2 |
| 13 | Diabetes with end-organ damage type1 and type2 | E10.2-E10.8  E11.2-E11.8 | 2 |
| 14 | Any tumor (except BC) | C00-C75 (excluding C50) | 2 |
| 15 | Leukemia | C91-C95 | 2 |
| 16 | Lymphoma | C81-C85; C88; C90; C96 | 2 |
| 17 | Moderate to severe liver disease | B15.0; B16.0; B16.2; B19.0; K70.4; K72; K76.6; I85 | 3 |
| 18 | Metastatic solid tumor | C76-C80 | 6 |
| 19 | AIDS | B21-B24 | 6 |

The Danish National Patient Registry covers all Danish Hospitals and has registered data on all non-psychiatric inpatient admissions and outpatient visits since 1977^3^.

# Table S3. SNP genotyping information, including observed and expected counts in total genotyping population (before restrictions to current study cohort).

| Gene | SNP ID | Bench-mark  MAF^b^ | TaqMan assay ID | Call rate  % | Minor allele | Frequency  % | Observed | | | | Expected | | | HWE  Chi^2^ |
| --- | --- | --- | --- | --- | --- | --- | --- | --- | --- | --- | --- | --- | --- | --- |
|  |  |  |  |  |  |  | Wildtype | Hetero-zygote | Homo-zygote | N/A | Wildtype | Hetero-zygote | Homo-zygote |  |
| *ABCB1* ^a^ | rs10248420 | 16% | C__30375194_10 | 64 | G | 2 | 3098 | 129 | 4 | 1856 | 3096 | 134 | 1 | 4.67 |
|  | rs1045642 | 47% | C___7586657_20 | 98 | G | 45 | 1508 | 2526 | 966 | 87 | 1536 | 2471 | 994 | 2.51 |
|  | rs1128503 | 44% | C___7586662_10 | 97 | A | 43 | 1652 | 2371 | 930 | 134 | 1626 | 2424 | 904 | 2.36 |
|  | rs2032582 | 46% | C_11711720C_30 | 96 | A | 45 | 1485 | 2373 | 1011 | 218 | 1466 | 2411 | 992 | 1.24 |
| *ABCC2* | rs12762549 | 48% | C__11214917_10 | 97 | G | 46 | 733 | 1138 | 544 | 69 | 702 | 1200 | 513 | 6.47 |
| *ABCG2* | rs2231142 | 11% | C__15854163_70 | 98 | A | 10 | 1961 | 446 | 25 | 53 | 1961 | 445 | 25 | 0.00 |
| *CYP1A1* ^a^ | rs1048943 | 4% | C__25624888_50 | 39 | C | 1 | 1972 | 20 | 10 | 3085 | 1962 | 40 | 0 | 490.45 |
| *CYP1B1* | rs1056836 | 43% | C___3099976_30 | 97 | C | 44 | 812 | 1093 | 508 | 72 | 461 | 1187 | 765 | 15.24 |
| *CYP3A* ^a^ | rs10273424 | 9% | C__29554473_10 | 95 | A | 8 | 4076 | 748 | 31 | 232 | 4079 | 742 | 34 | 0.27 |
| *CYP3A4* | rs2740574 | 4% | C___1837671_50 | 97 | G | 4 | 2231 | 177 | 12 | 64 | 2223 | 193 | 4 | 15.98 |
|  | rs35599367 | 5% | C__59013445_10 | 97 | T | 3 | 2251 | 144 | 8 | 82 | 2246 | 155 | 3 | 11.44 |
| *CYP3A5* ^a^ | rs776746 | 7% | C__26201809_30 | 100 | T | 8 | 4335 | 700 | 37 | 15 | 4328 | 715 | 30 | 2.22 |
| *GSTP1* | rs1138272 | 9% | C___1049615_20 | 99 | T | 8 | 2090 | 352 | 19 | 24 | 2086 | 359 | 15 | 0.96 |
| *SLCO1B1* | rs2306283 | 40% | C_1901697_20 | 96 | C | 41 | 860 | 1095 | 432 | 98 | 830 | 1155 | 402 | 6.47 |
|  | rs4149056 | 16% | C__30633906_10 | 96 | C | 14 | 1771 | 570 | 55 | 89 | 1764 | 584 | 48 | 1.28 |
| *SLCO1B3* | rs11045585 | 14% | C__31106434_10 | 98 | G | 14 | 1826 | 559 | 59 | 51 | 1814 | 583 | 47 | 4.22 |
| *ARHGEF10* | rs9657362 | 14% | C__25632922_10 | 90 | C | - | - | - | - | - | - | - | - | - |
| *EPHA4* | rs17348202 | 6% | C__34414779_10 | 98 | C | 5 | 2205 | 223 | 12 | 45 | 2199 | 234 | 6 | 1.28 |
| *EPHA5* | rs7349683 | 35% | C___1336545_30 | 99 | T | 35 | 1025 | 1116 | 307 | 37 | 1024 | 1118 | 305 | 0.01 |
| *EPHA6* | rs301927 | 16% | C___1037994_10 | 98 | G | 17 | 1665 | 673 | 86 | 61 | 1653 | 698 | 74 | 3.04 |
| *EPHA8* | rs209709 | 15% | C____702337_10 | 93 | G | - | - | - | - | - | - | - | - | - |
| *ERCC1* | rs11615 | 37% | C___2532959_1_ | 99 | G | 37 | 1118 | 991 | 341 | 35 | 1139 | 981 | 330 | 0.82 |
|  | rs3212986 | 25% | C_2532948_10 | 98 | A | 23 | 1441 | 869 | 121 | 54 | 1447 | 857 | 127 | 0.47 |
| *ERCC2* | rs13181 | 38% | C___3145033_10 | 97 | G | 37 | 1008 | 1025 | 371 | 81 | 962 | 1118 | 325 | 16.51 |
| *FGD4* | rs10771973 | 30% | C__30728517_30 | 99 | A | 29 | 1249 | 991 | 208 | 37 | 1243 | 1003 | 202 | 0.33 |
| *TRPV1* | rs879207 | 32% | C___1497993_10 | 93 | G | 32 | 1131 | 880 | 301 | 173 | 1067 | 1007 | 237 | 36.78 |

# ^a^ Genotyped in previous study.^4^

^b^ Minor allele frequencies in the non-Finnish European female population according to gnomAD.

# Table S4. Hazard ratios (HR) and 95% confidence intervals (CI) of return-to-work (RTW) and stable labor market attachment (SLMA) 0-6 months, 0-1 year, 0-2 years and 0-10 years surgery by genotypes in 21 SNPs.

|  | **0-6 months after surgery** | | **0-1 year after surgery** | | **0-2 years after surgery** | | **0-10 years after surgery** | |
| --- | --- | --- | --- | --- | --- | --- | --- | --- |
|  | **RTW**  **HR (95% CI)** | **SLMA**  **HR (95% CI)** | **RTW**  **HR (95% CI)** | **SLMA**  **HR (95% CI)** | **RTW**  **HR (95% CI)** | **SLMA HR (95% CI)** | **RTW**  **HR (95% CI)** | **SLMA HR (95% CI)** |
| ***ABCB1* rs1045642** |  |  |  |  |  |  |  |  |
| Any variants | 0.98 (0.79 - 1.23) | 1.00 (0.76 - 1.32) | 1.03 (0.91 - 1.18) | 1.02 (0.87 - 1.20) | 1.04 (0.94 - 1.16) | 1.04 (0.94 - 1.16) | 1.06 (0.96 - 1.17) | 1.04 (0.94 - 1.15) |
| Heterozygote | 0.96 (0.75 - 1.21) | 0.97 (0.72 - 1.29) | 1.01 (0.88 - 1.16) | 1.02 (0.86 - 1.21) | 1.05 (0.94 - 1.17) | 1.02 (0.91 - 1.15) | 1.06 (0.96 - 1.18) | 1.03 (0.93 - 1.15) |
| Homozygote | 1.06 (0.79 - 1.42) | 1.09 (0.76 - 1.56) | 1.08 (0.91 - 1.29) | 1.03 (0.83 - 1.28) | 1.04 (0.90 - 1.19) | 1.10 (0.95 - 1.27) | 1.04 (0.91 - 1.19) | 1.07 (0.93 - 1.22) |
| **ABCB1 rs2032582** |  |  |  |  |  |  |  |  |
| Any variants | 0.99 (0.80 - 1.24) | 1.02 (0.78 - 1.33) | 1.04 (0.92 - 1.18) | 1.00 (0.85 - 1.17) | 1.03 (0.93 - 1.14) | 0.96 (0.86 - 1.06) | 1.03 (0.94 - 1.14) | 0.98 (0.88 - 1.08) |
| Heterozygote | 0.99 (0.78 - 1.25) | 1.00 (0.76 - 1.33) | 1.06 (0.93 - 1.22) | 1.03 (0.87 - 1.21) | 1.06 (0.96 - 1.18) | 0.97 (0.87 - 1.08) | 1.06 (0.96 - 1.18) | 0.99 (0.89 - 1.10) |
| Homozygote | 1.01 (0.74 - 1.37) | 1.05 (0.73 - 1.52) | 0.99 (0.83 - 1.19) | 0.93 (0.74 - 1.16) | 0.95 (0.83 - 1.10) | 0.93 (0.80 - 1.08) | 0.96 (0.84 - 1.10) | 0.93 (0.81 - 1.07) |
| **ABCB1 rs2032582** |  |  |  |  |  |  |  |  |
| Any variants | 1.06 (0.85 - 1.32) | 1.06 (0.81 - 1.39) | 1.07 (0.94 - 1.22) | 1.04 (0.89 - 1.22) | 1.08 (0.97 - 1.19) | 1.02 (0.92 - 1.13) | 1.07 (0.97 - 1.18) | 1.03 (0.93 - 1.13) |
| Heterozygote | 1.03 (0.82 - 1.31) | 1.02 (0.76 - 1.36) | 1.10 (0.96 - 1.26) | 1.07 (0.90 - 1.26) | 1.11 (1.00 - 1.24) | 1.03 (0.92 - 1.15) | 1.11 (1.00 - 1.23) | 1.04 (0.94 - 1.16) |
| Homozygote | 1.12 (0.83 - 1.51) | 1.18 (0.83 - 1.69) | 1.01 (0.85 - 1.21) | 0.98 (0.78 - 1.22) | 0.98 (0.85 - 1.13) | 0.99 (0.86 - 1.15) | 0.98 (0.86 - 1.13) | 0.99 (0.86 - 1.13) |
| ***ABCC2* rs12762549** |  |  |  |  |  |  |  |  |
| Any variants | 1.08 (0.86 - 1.36) | 1.04 (0.79 - 1.36) | 1.02 (0.90 - 1.16) | 1.03 (0.88 - 1.21) | 0.99 (0.89 - 1.10) | 0.98 (0.88 - 1.09) | 1.00 (0.90 - 1.10) | 0.98 (0.89 - 1.08) |
| Heterozygote | 1.14 (0.90 - 1.45) | 1.10 (0.82 - 1.47) | 1.08 (0.94 - 1.24) | 1.08 (0.91 - 1.28) | 1.02 (0.92 - 1.14) | 1.01 (0.90 - 1.13) | 1.04 (0.93 - 1.15) | 1.01 (0.91 - 1.13) |
| Homozygote | 0.97 (0.72 - 1.30) | 0.91 (0.63 - 1.31) | 0.91 (0.77 - 1.09) | 0.92 (0.74 - 1.14) | 0.92 (0.81 - 1.05) | 0.93 (0.81 - 1.07) | 0.92 (0.81 - 1.05) | 0.92 (0.80 - 1.04) |
| \| ***ABCG2* rs2231142** \| \| --- \| |  |  |  |  |  |  |  |  |
| Any variants | 0.96 (0.74 - 1.25) | 1.18 (0.87 - 1.60) | 1.06 (0.91 - 1.24) | 1.06 (0.88 - 1.28) | 1.04 (0.92 - 1.17) | 1.06 (0.93 - 1.20) | 1.06 (0.95 - 1.20) | 1.06 (0.94 - 1.19) |
| Heterozygote | 0.98 (0.74 - 1.28) | 1.21 (0.88 - 1.65) | 1.04 (0.89 - 1.21) | 1.08 (0.89 - 1.30) | 1.01 (0.89 - 1.14) | 1.03 (0.91 - 1.18) | 1.04 (0.92 - 1.17) | 1.04 (0.92 - 1.17) |
| Homozygote | 0.72 (0.23 - 2.24) | 0.76 (0.19 - 3.05) | 1.48 (0.92 - 2.39) | 0.84 (0.40 - 1.77) | 1.60 (1.05 - 2.44) | 1.51 (0.97 - 2.35) | 1.61 (1.06 - 2.46) | 1.46 (0.95 - 2.24) |
| ***CYP1B1* rs1056836** |  |  |  |  |  |  |  |  |
| Any variants | 0.88 (0.71 - 1.09) | 0.87 (0.67 - 1.12) | 0.94 (0.83 - 1.07) | 1.01 (0.86 - 1.18) | 1.01 (0.92 - 1.12) | 1.02 (0.92 - 1.13) | 1.02 (0.93 - 1.13) | 1.05 (0.96 - 1.16) |
| Heterozygote | 0.79 (0.63 - 1.01) | 0.79 (0.59 - 1.06) | 0.93 (0.81 - 1.07) | 0.96 (0.81 - 1.14) | 1.00 (0.90 - 1.12) | 1.01 (0.90 - 1.13) | 1.01 (0.91 - 1.12) | 1.04 (0.94 - 1.16) |
| Homozygote | 1.06 (0.81 - 1.39) | 1.03 (0.73 - 1.44) | 0.97 (0.82 - 1.14) | 1.11 (0.91 - 1.36) | 1.04 (0.91 - 1.18) | 1.04 (0.91 - 1.20) | 1.06 (0.93 - 1.21) | 1.08 (0.95 - 1.23) |
| ***CYP3A* rs10273424** |  |  |  |  |  |  |  |  |
| Any variants | 0.89 (0.67 - 1.19) | 0.95 (0.67 - 1.34) | 1.14 (0.97 - 1.34) | 1.13 (0.93 - 1.37) | 1.14 (1.01 - 1.30) | 1.17 (1.03 - 1.33) | 1.15 (1.02 - 1.30) | 1.18 (1.04 - 1.33) |
| Heterozygote | 0.91 (0.68 - 1.22) | 0.96 (0.68 - 1.36) | 1.13 (0.96 - 1.32) | 1.10 (0.90 - 1.34) | 1.14 (1.00 - 1.29) | 1.16 (1.01 - 1.32) | 1.15 (1.01 - 1.30) | 1.17 (1.03 - 1.33) |
| Homozygote | 0.40 (0.06 - 2.83) | 0.61 (0.09 - 4.36) | 1.47 (0.76 - 2.84) | 1.93 (0.96 - 3.88) | 1.30 (0.74 - 2.30) | 1.52 (0.86 - 2.68) | 1.18 (0.67 - 2.08) | 1.32 (0.75 - 2.32) |
| ***CYP3A4* rs2740574** |  |  |  |  |  |  |  |  |
| Any variants | 1.31 (0.91 - 1.89) | 1.27 (0.81 - 1.99) | 1.07 (0.85 - 1.35) | 1.02 (0.77 - 1.36) | 1.00 (0.83 - 1.20) | 0.96 (0.79 - 1.17) | 0.97 (0.81 - 1.16) | 0.95 (0.79 - 1.13) |
| Heterozygote | 1.21 (0.83 - 1.79) | 1.08 (0.66 - 1.77) | 1.03 (0.81 - 1.30) | 0.93 (0.69 - 1.26) | 0.98 (0.81 - 1.19) | 0.94 (0.77 - 1.15) | 0.95 (0.79 - 1.15) | 0.92 (0.77 - 1.11) |
| Homozygote | 3.05 (1.14 - 8.18) | 5.13 (1.91 - 13.81) | 2.29 (1.02 - 5.10) | 3.76 (1.68 - 8.41) | 1.37 (0.65 - 2.87) | 1.50 (0.67 - 3.36) | 1.39 (0.69 - 2.78) | 1.51 (0.75 - 3.03) |
| ***CYP3A4* rs35599367** |  |  |  |  |  |  |  |  |
| Any variants | 1.05 (0.69 - 1.61) | 1.31 (0.82 - 2.10) | 0.98 (0.76 - 1.26) | 0.91 (0.66 - 1.25) | 0.99 (0.82 - 1.21) | 0.95 (0.77 - 1.17) | 0.98 (0.81 - 1.19) | 0.98 (0.81 - 1.19) |
| Heterozygote | 1.12 (0.73 - 1.70) | 1.38 (0.87 - 2.21) | 1.01 (0.78 - 1.30) | 0.94 (0.68 - 1.30) | 1.01 (0.82 - 1.23) | 0.97 (0.79 - 1.20) | 0.99 (0.81 - 1.20) | 0.99 (0.81 - 1.20) |
| Homozygote | N/A | N/A | 0.50 (0.12 - 2.00) | 0.39 (0.06 - 2.80) | 0.74 (0.31 - 1.79) | 0.63 (0.24 - 1.68) | 0.88 (0.39 - 1.95) | 0.90 (0.41 - 2.02) |
| ***CYP3A5* rs776746** |  |  |  |  |  |  |  |  |
| Any variants | 1.41 (1.07 - 1.85) | 1.49 (1.08 - 2.07) | 1.11 (0.93 - 1.32) | 1.13 (0.91 - 1.40) | 1.03 (0.89 - 1.18) | 1.03 (0.89 - 1.20) | 1.02 (0.89 - 1.16) | 1.01 (0.88 - 1.16) |
| Heterozygote | 1.48 (1.12 - 1.95) | 1.55 (1.11 - 2.15) | 1.15 (0.96 - 1.38) | 1.17 (0.94 - 1.45) | 1.09 (0.94 - 1.25) | 1.09 (0.94 - 1.27) | 1.07 (0.93 - 1.23) | 1.07 (0.93 - 1.23) |
| Homozygote | 0.37 (0.05 - 2.60) | 0.60 (0.08 - 4.27) | 0.55 (0.23 - 1.34) | 0.56 (0.18 - 1.73) | 0.42 (0.21 - 0.85) | 0.37 (0.17 - 0.82) | 0.48 (0.27 - 0.87) | 0.49 (0.27 - 0.88) |
| ***GSTP1* rs1138272** |  |  |  |  |  |  |  |  |
| Any variants | 1.00 (0.75 - 1.33) | 0.92 (0.64 - 1.32) | 0.94 (0.80 - 1.12) | 0.97 (0.78 - 1.19) | 0.92 (0.80 - 1.05) | 0.94 (0.82 - 1.08) | 0.94 (0.82 - 1.06) | 0.96 (0.85 - 1.10) |
| Heterozygote | 0.98 (0.73 - 1.32) | 0.87 (0.60 - 1.27) | 0.92 (0.77 - 1.09) | 0.93 (0.75 - 1.16) | 0.90 (0.78 - 1.03) | 0.92 (0.80 - 1.06) | 0.92 (0.80 - 1.05) | 0.94 (0.82 - 1.07) |
| Homozygote | 1.35 (0.43 - 4.21) | 2.02 (0.64 - 6.30) | 1.68 (0.90 - 3.12) | 1.79 (0.85 - 3.78) | 1.53 (0.87 - 2.70) | 1.85 (1.05 - 3.27) | 1.63 (0.95 - 2.82) | 1.94 (1.12 - 3.35) |
| ***SLCO1B1* rs2306283** |  |  |  |  |  |  |  |  |
| Any variants | 1.03 (0.83 - 1.27) | 0.95 (0.74 - 1.23) | 1.02 (0.90 - 1.16) | 1.01 (0.86 - 1.18) | 1.03 (0.93 - 1.14) | 0.99 (0.89 - 1.09) | 1.03 (0.93 - 1.13) | 0.99 (0.90 - 1.09) |
| Heterozygote | 1.06 (0.84 - 1.33) | 0.93 (0.70 - 1.23) | 1.03 (0.90 - 1.17) | 1.00 (0.85 - 1.19) | 1.04 (0.94 - 1.15) | 1.00 (0.89 - 1.11) | 1.04 (0.94 - 1.15) | 1.00 (0.90 - 1.11) |
| Homozygote | 0.95 (0.70 - 1.30) | 1.01 (0.71 - 1.45) | 1.00 (0.84 - 1.20) | 1.02 (0.82 - 1.26) | 1.01 (0.88 - 1.15) | 0.96 (0.83 - 1.11) | 1.00 (0.88 - 1.15) | 0.96 (0.84 - 1.10) |
| ***SLCO1B1* rs4149056** |  |  |  |  |  |  |  |  |
| Any variants | 1.02 (0.81 - 1.30) | 1.05 (0.79 - 1.40) | 1.05 (0.91 - 1.20) | 1.01 (0.85 - 1.20) | 1.04 (0.93 - 1.16) | 1.00 (0.90 - 1.13) | 1.03 (0.93 - 1.14) | 1.00 (0.90 - 1.11) |
| Heterozygote | 0.98 (0.77 - 1.26) | 1.02 (0.75 - 1.38) | 1.03 (0.89 - 1.19) | 1.01 (0.85 - 1.21) | 1.03 (0.92 - 1.15) | 1.00 (0.89 - 1.13) | 1.03 (0.92 - 1.14) | 1.00 (0.90 - 1.12) |
| Homozygote | 1.48 (0.81 - 2.70) | 1.37 (0.64 - 2.90) | 1.23 (0.82 - 1.83) | 0.98 (0.57 - 1.66) | 1.13 (0.82 - 1.57) | 1.01 (0.72 - 1.43) | 1.08 (0.79 - 1.49) | 0.97 (0.70 - 1.34) |
| **SLCO1B3 rs11045585** |  |  |  |  |  |  |  |  |
| Any variants | 1.08 (0.86 - 1.37) | 1.00 (0.75 - 1.34) | 1.02 (0.89 - 1.17) | 0.94 (0.79 - 1.12) | 1.03 (0.92 - 1.15) | 1.04 (0.92 - 1.16) | 1.01 (0.91 - 1.12) | 1.02 (0.91 - 1.13) |
| Heterozygote | 1.07 (0.83 - 1.37) | 0.95 (0.69 - 1.29) | 1.01 (0.88 - 1.17) | 0.91 (0.76 - 1.10) | 1.04 (0.93 - 1.17) | 1.03 (0.92 - 1.16) | 1.03 (0.92 - 1.15) | 1.02 (0.91 - 1.14) |
| Homozygote | 1.24 (0.68 - 2.27) | 1.52 (0.78 - 2.98) | 1.07 (0.73 - 1.56) | 1.19 (0.76 - 1.85) | 0.92 (0.67 - 1.26) | 1.09 (0.79 - 1.50) | 0.88 (0.65 - 1.19) | 0.96 (0.70 - 1.30) |
| **EPHA4 rs17348202** |  |  |  |  |  |  |  |  |
| Any variants | 1.44 (1.04 - 1.98) | 1.76 (1.23 - 2.54) | 1.08 (0.87 - 1.33) | 1.26 (0.99 - 1.62) | 1.03 (0.87 - 1.21) | 1.10 (0.92 - 1.30) | 1.02 (0.87 - 1.20) | 1.08 (0.92 - 1.27) |
| Heterozygote | 1.30 (0.92 - 1.83) | 1.65 (1.12 - 2.42) | 1.02 (0.82 - 1.27) | 1.20 (0.93 - 1.55) | 0.98 (0.83 - 1.17) | 1.06 (0.89 - 1.27) | 0.98 (0.83 - 1.16) | 1.04 (0.88 - 1.23) |
| Homozygote | 3.96 (1.76 - 8.88) | 3.68 (1.37 - 9.90) | 2.48 (1.23 - 4.97) | 2.61 (1.24 - 5.49) | 2.71 (1.45 - 5.05) | 2.18 (1.13 - 4.19) | 2.71 (1.45 - 5.05) | 2.32 (1.24 - 4.32) |
| ***EPHA5* rs7349683** |  |  |  |  |  |  |  |  |
| Any variants | 0.93 (0.75 - 1.15) | 1.00 (0.77 - 1.29) | 1.02 (0.90 - 1.15) | 1.02 (0.88 - 1.19) | 1.00 (0.91 - 1.10) | 1.04 (0.94 - 1.15) | 1.01 (0.92 - 1.11) | 1.03 (0.94 - 1.13) |
| Heterozygote | 0.98 (0.79 - 1.22) | 1.02 (0.78 - 1.34) | 1.02 (0.89 - 1.16) | 1.04 (0.89 - 1.23) | 0.98 (0.88 - 1.08) | 1.01 (0.91 - 1.13) | 0.99 (0.90 - 1.10) | 1.00 (0.91 - 1.11) |
| Homozygote | 0.77 (0.53 - 1.10) | 0.91 (0.60 - 1.38) | 1.02 (0.84 - 1.24) | 0.95 (0.74 - 1.21) | 1.09 (0.93 - 1.26) | 1.12 (0.95 - 1.31) | 1.10 (0.95 - 1.27) | 1.13 (0.98 - 1.31) |
| ***EPHA6* rs301927** |  |  |  |  |  |  |  |  |
| Any variants | 0.97 (0.77 - 1.22) | 0.97 (0.74 - 1.28) | 0.99 (0.87 - 1.13) | 0.92 (0.78 - 1.08) | 1.02 (0.92 - 1.13) | 0.97 (0.87 - 1.08) | 1.04 (0.94 - 1.15) | 0.99 (0.90 - 1.10) |
| Heterozygote | 0.99 (0.78 - 1.25) | 0.97 (0.72 - 1.29) | 1.02 (0.89 - 1.17) | 0.95 (0.80 - 1.12) | 1.03 (0.92 - 1.14) | 0.97 (0.86 - 1.08) | 1.04 (0.94 - 1.16) | 1.00 (0.90 - 1.11) |
| Homozygote | 0.84 (0.45 - 1.58) | 1.02 (0.50 - 2.07) | 0.80 (0.56 - 1.15) | 0.66 (0.40 - 1.09) | 0.98 (0.75 - 1.29) | 0.98 (0.74 - 1.29) | 0.99 (0.77 - 1.29) | 0.98 (0.76 - 1.28) |
| ***ERCC1* rs11615** |  |  |  |  |  |  |  |  |
| Any variants | 0.96 (0.78 - 1.19) | 0.98 (0.76 - 1.26) | 1.03 (0.91 - 1.17) | 0.95 (0.81 - 1.10) | 1.09 (0.99 - 1.20) | 1.08 (0.98 - 1.20) | 1.07 (0.98 - 1.18) | 1.07 (0.97 - 1.18) |
| Heterozygote | 1.02 (0.81 - 1.27) | 1.02 (0.78 - 1.34) | 1.07 (0.94 - 1.22) | 0.98 (0.84 - 1.16) | 1.10 (1.00 - 1.22) | 1.09 (0.98 - 1.22) | 1.08 (0.98 - 1.20) | 1.07 (0.97 - 1.18) |
| Homozygote | 0.80 (0.57 - 1.13) | 0.83 (0.55 - 1.26) | 0.91 (0.75 - 1.11) | 0.83 (0.65 - 1.07) | 1.06 (0.91 - 1.22) | 1.05 (0.90 - 1.23) | 1.05 (0.91 - 1.21) | 1.06 (0.92 - 1.23) |
| ***ERCC1* rs3212986** |  |  |  |  |  |  |  |  |
| Any variants | 0.83 (0.67 - 1.04) | 0.83 (0.64 - 1.08) | 0.94 (0.83 - 1.06) | 0.87 (0.75 - 1.02) | 1.05 (0.95 - 1.15) | 1.02 (0.92 - 1.13) | 1.04 (0.95 - 1.14) | 1.04 (0.94 - 1.14) |
| Heterozygote | 0.87 (0.69 - 1.08) | 0.84 (0.64 - 1.10) | 0.96 (0.84 - 1.09) | 0.90 (0.77 - 1.06) | 1.05 (0.95 - 1.17) | 1.03 (0.93 - 1.14) | 1.05 (0.95 - 1.15) | 1.04 (0.94 - 1.15) |
| Homozygote | 0.61 (0.34 - 1.09) | 0.76 (0.40 - 1.45) | 0.82 (0.61 - 1.11) | 0.65 (0.43 - 0.97) | 1.00 (0.80 - 1.24) | 0.98 (0.78 - 1.23) | 1.00 (0.81 - 1.24) | 1.02 (0.83 - 1.27) |
| ***ERCC2* rs13181** |  |  |  |  |  |  |  |  |
| Any variants | 0.89 (0.72 - 1.09) | 0.92 (0.71 - 1.18) | 0.99 (0.88 - 1.12) | 0.97 (0.83 - 1.12) | 1.03 (0.93 - 1.13) | 1.02 (0.93 - 1.13) | 1.02 (0.93 - 1.12) | 1.04 (0.94 - 1.14) |
| Heterozygote | 0.92 (0.74 - 1.15) | 0.98 (0.75 - 1.29) | 1.01 (0.89 - 1.15) | 0.97 (0.82 - 1.14) | 1.02 (0.92 - 1.14) | 1.01 (0.91 - 1.13) | 1.01 (0.92 - 1.12) | 1.03 (0.93 - 1.13) |
| Homozygote | 0.80 (0.57 - 1.10) | 0.74 (0.49 - 1.12) | 0.94 (0.78 - 1.14) | 0.96 (0.77 - 1.20) | 1.04 (0.90 - 1.20) | 1.06 (0.91 - 1.23) | 1.03 (0.90 - 1.18) | 1.08 (0.94 - 1.24) |
| ***FGD4* rs10771973** |  |  |  |  |  |  |  |  |
| Any variants | 0.91 (0.74 - 1.13) | 1.08 (0.83 - 1.39) | 0.96 (0.85 - 1.09) | 0.94 (0.81 - 1.10) | 0.97 (0.88 - 1.07) | 0.97 (0.88 - 1.07) | 0.98 (0.90 - 1.08) | 0.97 (0.88 - 1.06) |
| Heterozygote | 0.90 (0.72 - 1.13) | 1.05 (0.80 - 1.38) | 0.96 (0.85 - 1.09) | 0.94 (0.80 - 1.10) | 0.98 (0.88 - 1.08) | 0.98 (0.88 - 1.09) | 0.99 (0.90 - 1.09) | 0.98 (0.89 - 1.08) |
| Homozygote | 0.97 (0.66 - 1.42) | 1.19 (0.76 - 1.85) | 0.98 (0.78 - 1.23) | 0.94 (0.71 - 1.24) | 0.95 (0.79 - 1.14) | 0.94 (0.78 - 1.13) | 0.97 (0.82 - 1.15) | 0.92 (0.78 - 1.10) |

Abbreviations: CI= Confidence interval, HR=Hazard ratio, RTW= return-to-work, SLMA= stable labor market attachment.

**References**

1. Jensen VM, Rasmussen AW. Danish Education Registers. *Scand J Public Health*. 2011;39(7 Suppl):91-94. doi:10.1177/1403494810394715

2. Baadsgaard M, Quitzau J. Danish registers on personal income and transfer payments. *Scand J Public Health*. 2011;39(7_suppl):103-105. doi:10.1177/1403494811405098

3. Schmidt M, Schmidt SAJ, Sandegaard JL, Ehrenstein V, Pedersen L, Sørensen HT. The Danish National Patient Registry: a review of content, data quality, and research potential. *Clin Epidemiol*. 2015;7:449-490. doi:10.2147/CLEP.S91125

4. Ahern TP, Collin LJ, Baurley JW, et al. Metabolic Pathway Analysis and Effectiveness of Tamoxifen in Danish Breast Cancer Patients. *Cancer Epidemiol Biomark Prev Publ Am Assoc Cancer Res Cosponsored Am Soc Prev Oncol*. 2020;29(3):582-590. doi:10.1158/1055-9965.EPI-19-0833
